# Supplementary material for: Patterns of Intron Gain and Loss in Fungi
Source: PLoS Biol. 2004 Nov 30;2(12):e422. doi: 10.1371/journal.pbio.0020422 (PMC532390; doi:10.1371/journal.pbio.0020422)
Supplement: Table S1 — Also available at http://genes.mit.edu/NielsenEtAl/. (4.3 MB ZIP). [file pbio.0020422.st001.zip › NielsenEtAl/html/921.html]

AN5643.1.NCU03875.1.MG01012.1.FG10269.1


```
 CLUSTAL W (1.82) Multiple Sequence Alignments - Introns Inserted


Sequence 1: MG01012.1	1128 aa
Sequence 2: FG10269.1	1114 aa
Sequence 3: NCU03875.1	1127 aa
Sequence 4: AN5643.1	1111 aa
Alignment Length: 1139 aa
Number Identitical Residues: 709 aa
Alignment Score (without introns) 31780


MG01012.1 	MPRASRASA--AADTDASMVDAAPESAKRRGD--EM0EVD~ETPDYT~DSDTNPNTTASS
NCU03875.1	MAPRSRQP---VNDDDASMPDAP-ETSNAPAS--EM0EVD~ETPDYT~DSDTNPNTTESS
FG10269.1 	MAPRSRAN---AVDTDASMSDAQ---EHRQAE--DM0EVD~ETPDYT~DTE-NPSTTASS
AN5643.1  	MAPAAVPTSDPPSQADTPMTDANEEITSVPVDPLDA~KMT0DTPDYT0DSDTNPNTTASS
          	*.  :   :..  : *:.* **  .      .. :  ::  :***** *:::**.** **

MG01012.1 	VAGDLTV-DG~RKRRTEANQLRRSIFGRKHDRLGESK0EDDTLRRFRYLLGLTDLFRHFI
NCU03875.1	VAGEPVD-GH1CSHVSNFGPPPQLPGGLLFAILTRLQ~EDDSLRRFRYLLGLTDLFRHFI
FG10269.1 	VAGEPTG-DG~RRRRTEVNQLRRSIFGKKHDRLGESK0EDDTIRRFRYLLGLTDLFRHFI
AN5643.1  	VAGDVVPSDG~RRRRSEAFQMRKNMFGKKHGSLNENK0DDDSIRRFRYLLGLTDLFRHFI
          	***: . :.    : ::     :   *  .  * . : :**::*****************

MG01012.1 	ETNPDPNIRNIMEKIDAQNQEATKG--KKGASRQGGASSGRVRRTEAEEDAELLKDEKHG
NCU03875.1	ETNPNPKVREIMAEIDRQNAEDAKS--KKGAGRQGGATSDRRRRTEAEEDAELLKDEKVG
FG10269.1 	ETNPDPKIRDIMTEIDRQNAEAARA--KKGAGRQGGATSERRRRTEAEEDAELLKDEKQG
AN5643.1  	ESNPNPRIKEIMAEIDRQDAEEAAKSKRKVSARSGGASGERRRRTEQEEDAELLSDEKRG
          	*:**:*.:::** :** *: * :  :.:* :.*.***:. * **** *******.*** *

MG01012.1 	GSAETVFRESPAFIQGTMRDYQIAGLNWLISLHENGISGILADEMGLGKTLQTISFLGYL
NCU03875.1	GSAETVFRESPAFIQGTMRDYQIAGLNWLISLHENGISGILADEMGLGKTLQTISFLGYL
FG10269.1 	GSAETVFRESPSFVHGLMRDYQIAGLNWLISLHENGISGILADEMGLGKTLQTISFLGYL
AN5643.1  	GGTNTIFRESPPFIQGEMRDYQIAGLNWLVSLHENGISGILADEMGLGKTLQTISFIGYL
          	*.::*:*****.*::* ************:**************************:***

MG01012.1 	RHIMGITGPHLVIVPKSTLDNWKREFGKWTPEVNVLVLQGAKEERAALIAERLVDESFDV
NCU03875.1	RHIMDTPGPHLVIVPKSTLDNWKREFAKWTPEVNVLVLQGAKEERQQLIQDRLVDENFDV
FG10269.1 	RHILDITGPHLVIVPKSTLDNWKREFAKWTPEVDVLVLQGAKDERQALINDRLVDEKFDV
AN5643.1  	RHLCDITGPHLVAVPKSTLDNWKREFHKWTPEVNVLVLQGDKEERHKLINERLLDEDFDV
          	**: . .***** ************* ******:****** *:**  ** :**:**.***

MG01012.1 	CITSYEMILREKSHLKKFAWEYIIIDE~AHRIKNEESSLAQVIRLFNSRNRLLITGTPLQ
NCU03875.1	CITSYEMILREKAHLKKFAWEYIIIDE~AHRIKNEESSLAQVIRVFNSRNRLLITGTPLQ
FG10269.1 	CITSYEMVLREKSHLKKFAWEYIIIDE0AHRIKNEESSLSQVIRLFDSRNRLLITGTPLQ
AN5643.1  	CITSYEMILREKSHLKKFAWEYIIIDE~AHRIKNEESSLAQIIRVFNSRNRLLITGTPLQ
          	*******:****:************** ***********:*:**:*:*************

MG01012.1 	NNIHELWALLNFLLPDVFGDSEAFDQWFSGEGQDSDTVVQQLHRVLRPFLLRRVKADVEK
NCU03875.1	NNLHELWALLNFLLPDVFGDSEAFDQWFSGQDRDQDTVVQQLHRVLRPFLLRRVKSDVEK
FG10269.1 	NNLHELWALLNFLLPDVFGDSEAFDQWFSGQDRDQDTVVQQLHRVLRPFLLRRVKSDVEK
AN5643.1  	NNLHELWALLNFLLPDVFGDSEAFDQWFSNQEADQDTVVQQLHRVLRPFLLRRVKSDVEK
          	**:**************************.:  *.********************:****

MG01012.1 	SLLPKKEVNLYLKMTEMQRTWYQKILEKDIDAVNGANGKRESKTRLLNIVMQLRKCCNHP
NCU03875.1	SLLPKKEVNVYIGMSEMQVKWYQKILEKDIDAVNGAGGKRESKTRLLNIVMQLRKCCNHP
FG10269.1 	SLLPKKEVNVYLGMSEMQVKWYQKILEKDIDAVNGAGGKRESKTRLLNIVMQLRKCCNHP
AN5643.1  	SLLPKKEVNLYVPMSSMQVKWYQKILEKDIDAVNGAGGKKESKTRLLNIVMQLRKCCNHP
          	*********:*: *:.** .****************.**:********************

MG01012.1 	YLFEGAEPGPPYTTDEHLVYNSGKMVVLDKLLKRLKAQGSRVLIFSQMSRVLDILEDYCV
NCU03875.1	YLFEGAEPGPPYTTDEHLVYNAGKMLVLDKLLKRLQKQGSRVLIFSQMSRLLDILEDYCV
FG10269.1 	YLFEGAEPGPPYTTDEHLVYNAGKMAVLDKLLKRLQKQGSRVLIFSQMSRLLDILEDYCV
AN5643.1  	YLFEGAEEGPPYTNDVHIINNSGKMVILDKLLARMQAQGSRVLIFSQMSRVLDILEDYCA
          	******* *****.* *:: *:*** :***** *:: *************:********.

MG01012.1 	FREYKYSRIDGGTAHEDRIAAIDEYNKPGSEKFVFLLTTRAGGLGINLTTADIVILFDSD
NCU03875.1	FRQYKYCRIDGGTAHEDRIAAIDEYNKPDSDKFVFLLTTRAGGLGINLTSADIVILYDSD
FG10269.1 	FREYKYCRIDGGTAHEDRIAAIDEYNKPGSEKFVFLLTTRAGGLGINLTTADIVVLYDSD
AN5643.1  	LRKYQYCRIDGTTAHEDRIAAIDEYNKPDSDKFIFLLTTRAGGLGINLTTADIVVLYDSD
          	:*:*:*.**** ****************.*:**:***************:****:*:***

MG01012.1 	WNPQADLQAMDRAHRIGQTKQVYVYRFLVDNTIEEKVLERAAQKLHLDRLVIQQGRAQVA
NCU03875.1	WNPQADLQAMDRAHRIGQTKQVVVYRFVTDNAIEEKVLERAAQKLRLDQLVIQQGRAQIA
FG10269.1 	WNPQADLQAMDRAHRIGQTKQVVVYRFVTENAIEEKVLERAAQKLRLDQLVIQQGRAQQA
AN5643.1  	WNPQADLQAMDRAHRIGQTKQVVVYRFITESAIEERVLERAAQKLRLDQLVIQQGRAQQQ
          	********************** ****:.:.:***:*********:**:*********  

MG01012.1 	AKAAANKDELLSMIQHGAAKVFN-KDSDGDVVKSADDIQDDDIEAMLASGESRTRELNAR
NCU03875.1	AKAAANKDELLSMIQHGAEKVFQSKGGFGALPENGAELGDDDIDAILAAGENRTKELNAK
FG10269.1 	AKAAANKDELLSMIQHGAEKVFQSKGATGTMGSKDGEVDDDDIDAILARGEDRTKELNAK
AN5643.1  	AKNTASKDELLGMIQHGAAEIFNKQGGTSTLQE-GKDITDDDIDAILRKGEERTAELSKK
          	** :*.*****.****** ::*:.:.. . : .   :: ****:*:*  **.** **. :

MG01012.1 	YEKLGIDDLQKFSSESAYDWNGENFALVNKSKDIGANWINPAKRERKEQTYSVDKYYRQA
NCU03875.1	YEKLGLDDLQKFTSESAYEWNGEDFAARKKD--IGINWINPAKRERKEQIYSIDKYYKQA
FG10269.1 	YEKLGIDDLQKFTSESAYEWNGENFANTKKN--INTTWINPAKRERKEQSYSMDKYFRQT
AN5643.1  	YEQLGIDDLQKFNSESAYEWNGKDFTTDKKK-DIGIHWINPAKRERKEQFYSIDKYYRQA
          	**:**:******.*****:***::*:  :*. .*.  ************ **:***::*:

MG01012.1 	IYGHTGQPKEVKPKAPRPPKQVHIQDYQFFPPELRDLQDRETAFYRKEIGYKVPLPDGDD
NCU03875.1	LQTGG-RTADTKPKAPRAPKQVPVHDYQFYPLRLRELQDRETAYYRKEIGYKVPLPDGDD
FG10269.1 	MYPNP--KADAKPKAPRAPKQVPVHDYQFYPPRLRDLQDRETAYYRKEIGYKVPLADGEE
AN5643.1  	LATGG-RTADPKPKVPRAPKQIAIHDWQFFPPGLQELQDKETAYFHKEIGYKVPLPDGPE
          	:        : ***.**.***: ::*:**:*  *::***:***:::*********.** :

MG01012.1 	ATLSDREAEQALDQQEIDNATPLTEEEQQRKQELSEEGFSEWNRRDFQQFVNGCHKFGRN
NCU03875.1	ETVEEREAERALDQQEIDNATPLTEEEQAEKQKLAEQGFGDWNRRDFQQFINGSGRYGRN
FG10269.1 	DTLEEREAERALDQQEIDNATPLTEEEREEKETLSLQGFGDWNKRDFQQFVNGSGKYGRH
AN5643.1  	E-LTHREAERDLEQAMIDNAAPLTEAEQAEKTKLSEQGFSDWNRRDFQQFINGSAKFGRT
          	  : .****: *:*  ****:**** *: .*  *: :**.:**:******:**. ::** 

MG01012.1 	DFEGIATEVVGKTAAEIKAYSKVFWQRYTEIADYHKYIKTIEDGEEKLRKIEHQRKMLRK
NCU03875.1	DYEGIAQEVDSKTPAEIKAYAKVFWQRYTEIADYNKYLKVIEDGEERMRKIEHQRKMLRK
FG10269.1 	EYEGISQEIDSKSPPEIKAYAKVFWQRYTEIADYPKYIKTIEDGEERTRRIEHHQKLLKK
AN5643.1  	DYAGIATEVDSKDPKQVEEYANVFWKRYTEIQDYPKYIRVIEQGEEKLRKMGHQRKMLRK
          	:: **: *: .* . ::: *::***:***** ** **::.**:***: *:: *::*:*:*

MG01012.1 	KMSQYRVPLQQLKINYSVSTTNKKVYTEEEDRFLLVQLDKHGIDSDGIFETIRDEIRDSA
NCU03875.1	KMSQYRVPLQQLKINYTVSTTNKKVYTEEEDRFLLVMLDKYGVDSEGIYEKIRDEIRESP
FG10269.1 	KMGQYRVPLQQLKINYSVSTTNKKVYTEEEDRFLLVLLDRYGIDSEGLYEKMRDDIRESP
AN5643.1  	KMEMYRVPLQQLKINYTVSTTNKKVYTEEEDRFLLVMLDKYGVEGEGLYEKIREEVRESP
          	**  ************:******************* **::*::.:*::*.:*:::*:*.

MG01012.1 	LFRFDWFFLSRTPTELSRRATTLLTTIVKEFDDMN--TTKGTNGTANGRAKRDVDDEEND
NCU03875.1	LFRFDWFFLSRTPIEISRRCNTLLTTIVKEFEDEPSNTTKAKVNGVNGKLKREANDEDND
FG10269.1 	LFRFDWFFLSRTPIELSRRCTTLITTIVKEFEDVP--ARNGVN----GKSKREPDDE-ND
AN5643.1  	LFRFDWFFLSRTPVEIGRRCTTLLNTIAKEFEPDG------KNGDGKGRGRDREDDELDN
          	************* *:.**..**:.**.***:           .  .*: :   :** ::

MG01012.1 	EDSILGMAPPKKKAKN-GVK0NKT~LDNVKSTTASKNNSAAPSRASSVNSNTSPAPPAKS
NCU03875.1	EDSVLGLAPAKKKTKANGVK0NKA~LDNVKSASGSKATSAAPSRASSVASTESKATKGKA
FG10269.1 	EDSILGMAPAKKKAKN-EVK0NKA~LDNVKSVKSSKNSSATPSRASSVASTASAGGSAKS
AN5643.1  	ED----DVPAKKKTKG----~-AV0NKQVKAVKGSKGNSASTSRASSANPPKS-------
          	**     .*.***:*        .  .:**:...** .**:.*****. .  *       

MG01012.1 	KAKGKKK
NCU03875.1	APKGKKK
FG10269.1 	K-KGRKK
AN5643.1  	--RGRKK
          	  :*:**
```
